# A Robust Kalman Algorithm to Facilitate Human-Computer Interaction for People with Cerebral Palsy, Using a New Interface Based on Inertial Sensors

**DOI:** 10.3390/s120303049

**Published:** 2012-03-06

**Authors:** Rafael Raya, Eduardo Rocon, Juan A. Gallego, Ramón Ceres, Jose L. Pons

**Affiliations:** Bioengineering Group, CSIC, Crta. Campo Real Km 0.2 La Poveda, Arganda del Rey, Madrid 28500, Spain; E-Mails: e.rocon@csic.es (E.R.); gallego@iai.csic.es (J.A.G.); ceres@iai.csic.es (R.C.); jlpons@iai.csic.es (J.L.P.)

**Keywords:** inertial sensors, human-computer interface, cerebral palsy, Kalman filter

## Abstract

This work aims to create an advanced human-computer interface called ENLAZA for people with cerebral palsy (CP). Although there are computer-access solutions for disabled people in general, there are few evidences from motor disabled community (e.g., CP) using these alternative interfaces. The proposed interface is based on inertial sensors in order to characterize involuntary motion in terms of time, frequency and range of motion. This characterization is used to design a filtering technique that reduces the effect of involuntary motion on person-computer interaction. This paper presents a robust Kalman filter (RKF) design to facilitate fine motor control based on the previous characterization. The filter increases mouse pointer directivity and the target acquisition time is reduced by a factor of ten. The interface is validated with CP users who were unable to control the computer using other interfaces. The interface ENLAZA and the RKF enabled them to use the computer.

## 1. Introduction

### 1.1. Definition and Classification of Cerebral Palsy

Cerebral palsy (CP) is the most common motor disability in childhood and involves a disorder of movement, posture and motor function. It is caused by a non-progressive interference, lesion, or abnormality in the immature, developing brain. CP involves a group of disorders that are permanent but not unchanging [1]. The prevalence of CP is internationally 1.5–2.0 cases per 1,000 births. More than 500,000 people in the United States have CP [2]. In Europe these figures are even higher [3]. CP is an umbrella term that involves a wide variety of diseases. It can be classified according to the pathology of brain injury or according to the timing of brain injury. The “Surveillance of cerebral palsy in Europe (SCPE): a collaboration of cerebral palsy surveys and registers” presented a consensus on the definition, classification and description of CP [4,5]. The classification based on predominant neuromotor abnormality divides CP into: spastic, dyskinetic or ataxic, with dyskinesia further differentiated into dystonia and choreoathetosis. The changing nature of symptoms and signs makes the clinical classification difficult in the first years of life as the pattern of movement and muscle tone may change completely.

The WHO International Classification of Functioning, Disability and Health (ICF) along with several other recent publications have sensitized health professionals to the importance of evaluating the functional consequences of different health states. The SCPE also recommended describing functional severity in legs and arms according to standardized scores. For ambulation, the Gross Motor Function Classification System (GMFCS) [6] has been widely employed internationally to group individuals with CP into one of five levels based on functional mobility or activity limitation. So has the bimanual fine motor function system BFMF [7], or, in prospective studies, the Manual Ability Classification System MACS [8].

### 1.2. Human-Computer Interfaces for People with CP

People with CP often have severe limitations using conventional human-computer interfaces (HCI), thus diminishing their opportunities to communicate and learn through computers [9]. Davies *et al*. presented a systematic review of the development, use and effectiveness of devices and technologies that enable or enhance self-directed computer access by individuals with CP [10]. They divided HCI into five categories:

- pointing devices,
- keyboard modifications,
- screen interface options,
- speech and gesture recognition software and
- algorithms and filtering mechanisms

Touch screens [11], switches with scanning approaches [9] and joysticks [12] are examples of the first category. Man *et al*. [9], presented a study to compare four different computer-access solutions: the *CameraMouse*, (head tracking using a webcam [13]), the *ASL Head Array*, (a mouse emulator using head switches), the *CrossScanner* (1–2 switch mouse emulator), and the *Quick Glance Eye Tracking System* (eye tracking using infrared sensors). Two students with quadriplegic CP with dyskinetic athetosis participated.

The *CrossScanner* showed the highest rate of accuracy among the four systems and across the two participants. The *CameraMouse* was considered an attractive tool for postural training. The capture field of the *Quick Glance Eye Tracking System* is limited by the transmission angle of the infrared light. The participants had athetosis, which literally means “without fixed position” [14]. *Quick Glance Eye* showed low performance because the subjects continually moved out of the capture field.

Eye and face tracking interfaces are powerful pointing devices for people with motor disorders. They often succeed in improving human-computer interaction [15]. They have the potential to be a very natural form of pointing, as people tend to look at the object they wish to interact with. However, they often present low performance with people with severe motor disorders.

As regards keyboard-based solutions, some studies have demonstrated that keyboard adaptations improve speed and accuracy [16,17]. The category “screen interface options” includes the interfaces that scan through screen icons or dynamically change the icon position. Children with significant physical impairments (who are unable to point) use visual scanning and switches to select symbols. Symbol-prediction software is a method of access that involves highlighting a specific symbol within an array on the basis of an expected or predicted response [18]. The prediction software reduces the response time required for participants but there is a trade-off between speed and accuracy.

Some devices are voice-based human-computer interfaces in which a set of commands can be executed by the voice of the user. Speech-recognition software is difficult to customize for users with CP who have dysarthric speech. A combination of feedback information through auditory repeat and visual feedback may help users to reduce variability in dysarthric utterances and enable increased recognition by speech-recognition software [19,20].

Algorithms and filtering mechanisms are focused on improving the accuracy of computer recognition of keyboard input or tracking of the pointer motion. In connection with the techniques to facilitate pointer control, there are two different approaches: (1) *target-aware* and (2) *target-agnostic*. Target-aware techniques require the mouse cursor to know about and respond to the locations and dimensions of on-screen targets. Examples are gravity wells [21], force fields [22] and bubble cursors [23]. In contrast, few techniques are target-agnostic, meaning that the mouse cursor can remain ignorant of all on-screen targets, and targets themselves are not directly manipulated. Conventional pointer acceleration is by far the most common target-agnostic technique found in all modern commercial systems. Wobbrock *et al*. [24] have designed an algorithm that adjusts the mouse gain based on the deviation of angles. People with CP and other disabilities (e.g., Parkinson’s or Friedreich’s ataxia) participated. The authors concluded that the algorithm improved pointing throughput by 10.3% over the Windows default mouse and 11% over sticky icons. Some mathematical analyses showed that additional modeling and filters within the computer software could theoretically improve icon selection using a mouse as the input, but this was not tested in real time [25].

Access solutions for individuals with CP are in the early stages of development and future work should include assessment of end-user comfort, effort and performance, as well as design features [10]. A fundamental conclusion is that there is a wide diversity of solutions but their authors frequently assert that usability decreases dramatically when users have a severe motor disability.

### 1.3. Motivation and Objective

Human-machine interaction is often limited by the disability caused by CP. There are many technologies that can be useful to create alternative interfaces. This work presents an adaptive algorithm to reduce the effect of involuntary movements on human-machine interaction. This aim can be achieved as follows:

- characterizing the motor disorders in terms of motor control, frequency and range of motion (ROM). This work was presented in [26] (summarized in Section 3),
- designing the filtering algorithm based on the previous characterization (Section 4),
- validating the filtering algorithm with people with CP in real time (Section 5).

## 2. A New Interface Based on Inertial Sensors: The ENLAZA Interface

**Figure 1.**
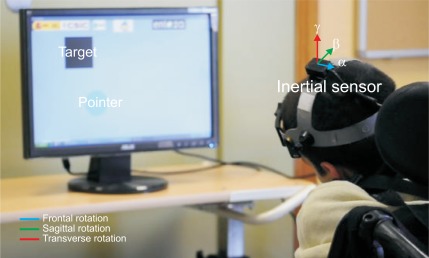
Experiments with the inertial interface at Cantabria ASPACE (Spain).

The ENLAZA interface is based on inertial technology. The inertial measurement unit (IMU), developed jointly by the authors and Technaid S.L., integrates a three-axis gyroscope, a three-axis accelerometer and a three-axis magnetometer. The rate gyroscope measures angular velocity by measuring capacitance based on the Coriolis force principle. The accelerometer measures the gravity and the acceleration caused by motions (by Hookes law). The magnetometer measures the Earth’s magnetic field. The 3D IMU is based on microelectromechanical systems (MEMS) and is available in a package measuring 27 mm × 35 mm × 13 mm and it weighs 27 g, which is less than other sensors used in the field [27,28]. The 3D IMU is capable of sensing ±2.0 Gauss, ±500°*/s* angular rate and ±3 g acceleration about three axes independently. It has an angular resolution of 0.05°, a static accuracy less than 1° and a dynamic accuracy about 2° RMS. The interface consists of a headset with a commercial helmet and an IMU. Figure 1 depicts a person with CP using the inertial interface.

The IMU is used to estimate the sagittal and transverse rotations that are translated to pointer positions by means of a calibrated range of motion. These rotations can be calculated by the Euler angles using the equations:

(1) $$\begin{array}{lll} R_{\mathrm{GS}} & = & R_{s}\cdot {\left(R_{G}\right)}^{-1}\\ \alpha & = & \text{arctan}(-R_{\mathrm{GS}}(2,\;3)/R_{\mathrm{GS}}(3,\;3))\\ \beta & = & \text{arcsin}(R_{\mathrm{GS}}(1,\;3))\\ \gamma & = & \text{arctan}(-R_{\mathrm{GS}}(1,\;2)/R_{\mathrm{GS}}(1,\;1)) \end{array}$$

where *R_G_* is the rotation matrix during calibration (global reference corresponding to the center of the screen). *R_s_* is the rotation matrix during the task. The *β* and *γ* angles (sagittal and transverse rotations respectively) are translated to vertical and horizontal pointer positions. The frontal rotation (*α*) does not cause any displacement.

The inertial interface was validated by healthy users [29]. The metric used was the *Throughput* that is defined by the standard “*ISO 9241-Part 9. Requirements for non-keyboard input devices*” to assess the usability of the HCI. The *Throughput* is specifically described for target-reaching tasks. It is assumed to be a reliable estimation of goal-directed motor coordination. The metric is based on Fitts’s law that models human psychomotor behavior based on Shannon’s Theorem [30]. Human psychomotor behavior is simulated as a channel for information transmission measured in bits/s. A signal is transmitted through a non-ideal medium and is perturbed by noise. The effect of the noise is to reduce the information capacity of the channel. Fitts’s law proposes a logarithm-linear relationship between the amplitude of the movement, the target width and the average movement time (Equation (2)).

(2) $$T=a+{\mathrm{blog}}_{2}(1+\frac{D}{W})$$

where *T* is the average time taken to complete the movement, *a* is the intercept and defines delay in the psychomotor system or reaction time and *b* is the slope coefficient. These constants can be determined experimentally by fitting a straight line to measured data. *D* is the distance from the starting point to the center of the target and *W* is the width of the target measured along the axis of motion. The ENLAZA interface was validated on 5 healthy participants using the protocol presented in [29]. The average *Throughput* was about 2 bits/s. This result is in agreement with the literature. Table 1 shows the *Throughput* values for similar interfaces.

## 3. Characterizing CP Motor Disorders

### 3.1. Objective

The characterization of abnormal head movements was presented in [26]. This section presents a summary of this characterization because it is the basis of the filter design.

### 3.2. Involuntary Movement and Posture

Head movement may be affected by any of the five basic types of dyskinesia: tremor, tic, chorea, myoclonus, and dystonia [35,36]. In addition, the head is subject to two dyskinesias that we call “flopping” and “nodding”. Head tremor is an active, wholly involuntary, sustained pendular oscillation. Myoclonus can either be jerks (rapid contractions) or rhythmical (resembling tremor). Flopping is a passive, involuntary movement characterized by transient, exponentially decaying occurring at the end of active head movement. Tic and nodding are acquired behavioral patterns. A tic is a single, rapid, stereotyped movement. Nodding is an active, regular, sustained, usually pendular oscillation.

### 3.3. Main Outcome Measures

The main outcome measures for characterization were selected according to three domains: time, frequency and space.

1. Time-domain analysis. Motor disorders can be evidenced by muscle tone variations, causing difficulties in fulfilling the speed-accuracy trade-off stated by Fitts’s law. The metric for the time-domain measures the correlation between the voluntary psychomotor model and the captured data (R-squared, R2).
2. Frequency-domain analysis. A frequency-domain analysis is necessary because motor disorders are frequency- and time-varying. Components calculated from Fast Fourier Transform (FFT) are (1) frequency at peak amplitude and (2) frequency corresponding to the first 75% of the area of the spectral energy. A low-pass filter at a cutoff frequency of 10 Hz was introduced to reduce the influence of electrical noise on the measurement. This frequency leaves the movement unaltered, because most voluntary and involuntary movements range from 0 to 4 Hz [36].
3. Abnormal postures can be identified by measuring the spatial variables such as predominant head orientation and ROM. Neck/head motion is clinically described as rotations about 3 orthogonal axes embedded in the head. Euler angles are useful for describing human motion such as head movement because they define rotation using three angles that can easily be physically related to frontal, sagittal, and transverse (*α*, *β*, *γ*) axes that are calculated using Equation (1). ROM is defined as the difference between the maximum and minimum values of *α*, *β* and *γ*. We calculate the ratio between the range needed to reach a target and the total range. If the ratio is less than 1, the subject moved his/her head between the screen limits; otherwise the head was out of range. *α* is given as absolute value because frontal rotation does not produce pointer displacements.

### 3.4. Participants

Four people with severe CP were recruited (Table 2). Their mean age was 29 years (range: 26–35). They were unable to use the mouse pointer or the keyboard. Three healthy users participated to extract the normalized patterns for comparison. Patient trials were carried out at Cantabria ASPACE, a specialized CP center with expertise in using alternative human-computer interfaces. Tests with healthy users were carried out at Bioengineering Lab-CSIC (Madrid, Spain). Experiments and protocols were approved by the Cantabria ASPACE expert committee.

### 3.5. Methods

Participants were instructed to locate the cursor over the target as quickly as possible using head motion. The target then changed its position, following a sequential order. One session consisted of reaching 15 targets. There were 5 sessions per user during a week, thus the target-reaching task was carried out 75 times in total. The target-reaching task is attractive because it provides a statistical description of the involuntary movements made during voluntary activity.

### 3.6. Results

The time-domain analysis showed relevant information about voluntary behavior during a reaching task. The reaching task can be modeled by:

1. an initial movement that rapidly covers distance and
2. a slower homing-in phase.

This voluntary movement describes a log-linear law between amplitude of movement and time in order to maintain the trade-off between speed and accuracy. The motion can consist of several “sub-movements” especially to home on the target. The “sub-movements” following the initial movement are usually performed with lower velocity and correspond to the trajectory correction. The R2 correlation between Fitts’s model and psychomotor behavior was about 83% for the healthy subjects. The CP participants had lower correlation compared to the healthy users, implying that they had more difficulties in maintaining the speed-accuracy trade-off. The correlation was lower for CP1 (32%) with respect to the remaining users (50–75%). Gross motor control was better than fine motor control because there was an initial movement that rapidly covers distance. However, there were many overshoots and undershoots causing many sub-movements around the target.

The frequency-domain analysis showed that the predominant component for the healthy users was about 0.3 Hz. Three-quarters of total amplitude frequency ranged between 1.5 Hz and 3.5 Hz with a mean about 2 Hz (75% of the total power spectral density ranges from 0 to 2 Hz [26]). This result has been found to be in agreement with the literature [36]. Analyzing the movement of the people with CP, an important conclusion can be obtained: voluntary and involuntary movements share the same bandwidth. This fact must be considered to design the filtering technique. Nevertheless, the movement of CP1 presented a higher predominant frequency (*>*1 Hz) compared to the rest of the users with CP due to the athetotic movements associated with hypertonia. Dystonia (CP2 and CP4) causes jerky movements with irregular amplitudes and variable frequency. These results are dissimilar to other motor disorders such as Parkinson‘s resting tremor because the frequency of the tremor is relatively constant in any one patient in the range of 3–7 Hz. Table 3 summarizes the frequency data.

The spatial-domain analysis showed that the healthy users had good postural control. The range of motion offers useful information to evaluate postural stability. The ROM analysis for people with CP revealed a meaningful difference for hypotonia. Hypotonia is a decreased muscle tone that causes the head to drop forward. Sagittal ROM is more unbalanced due to the gravity effect.

According to the analysis in time, frequency and spatial domain, the following conclusions can be obtained:

- Hypertonia and athetosis movements cause involuntary movements at higher peak frequency than voluntary movements.
- Hypotonia is characterized by abnormal postural activity. The frequency of hypotonic movements is similar to the frequency of voluntary motion.
- Voluntary- and involuntary-movement frequencies share the same bandwidth.
- The spatial-domain analysis showed that the people with CP had a higher difficulty (greater difficulty) for postural control in the sagittal plane. This result was especially observed for hypotonic cases because of the low muscle tone and the pull of gravity.
- Time-domain analysis revealed that fine motor control is more affected than gross motor control.

Table 4 summarizes the design principles of the filtering technique based on motor and posture characterization.

## 4. Filtering Algorithms

**Figure 2.**
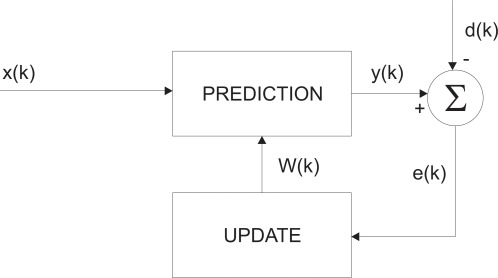
Adaptive-filtering block diagram.

The proposed hypothesis states that long trajectories of the pointer correspond to voluntary movements whereas rapid changes are involuntary. As a consequence, the filter should dynamically adapt its gain according to trajectory deviations. An adaptive filter is time-varying since its transfer function is continually adjusted and driven by a reference signal that depends on the application. The general adaptive-filtering block diagram consists of the prediction and update steps as depicted in Figure 2. The parameter *k* is the iteration number, *x*(*k*) denotes the input signal, *y*(*k*) is the output signal and *d*(*k*) defines the desired signal. The error signal *e*(*k*) is the difference between d(k) and y(k). The filter coefficients *W* (*k*) are updated as stated by the error signal.

The equation parameters can be adjusted to track the movements of the mouse pointer. Some works assume a constant velocity model to describe the dynamics of the mouse pointer [37]. This assumption is reasonable when that sample period is very small compared with the movement speeds [38]. In our case, the sample period adopted was 20 ms and the dominant frequency of voluntary movement is 0.3 Hz meaning that this assumption is adequate.

The Benedict–Bordner filter [39] and the Kalman filter are adaptive filters commonly used in tracking applications. These algorithms were successfully applied by some authors for tremor suppression [40–42]. The purpose of this investigation is to determine the feasibility of using these adaptive filters for reducing the motor disability effects caused by CP. In addition, we propose a *robust Kalman filter* that theoretically improves the performance of the classic Kalman in the presence of data outliers.

### 4.1. Benedict–Bordner Filter (BBF)

The g–h filter (sometimes called *α*-*β* filter) is a simple recursive adaptive filter assuming that velocity remains approximately constant. It is used extensively as a tracking filter. The g–h algorithm consists of a set of update equations:

(3) $${\dot{x}}_{k,k}^{*}={\dot{x}}_{k,k-1}^{*}+h_{k}\;\left(\frac{y_{k}-x_{k,k-1}^{*}}{T}\right)$$

(4) $$x_{k,k}^{*}=x_{k,k-1}^{*}+g_{k}\;\left(y_{k}-x_{k,k-1}^{*}\right)$$

and prediction equations [38]:

(5) $$x_{k+1,k}^{*}=x_{k,k}^{*}$$

(6) $$x_{k+1,k}^{*}=x_{k,k}^{*}+T{\dot{x}}_{k+1,k}^{*}$$

The tracking update equations or estimation equations (Equations (3) and (4)) provide the mouse pointer speed and position. The predicted position is an estimation of *x_k_*_+1_ based on past states and prediction, Equations (5) and (6), and it takes into account current measurement using updated states. *T* is the sample period. The selection of the parameters *g* and *h* determines whether we put the combined estimate closer to *y_k_* or to $x_{k,k-1}^{*}$.

The Benedict–Bordner estimator is designed to minimize the transient error. Therefore, it responds faster to changes in movement speed and is slightly under damped [43]. The relation between filter parameters is defined by Equation (7):

(7) $$h=\frac{g^{2}}{2-g}$$

g–h gains are manually selected and static.

### 4.2. Kalman Filter (KF)

**Figure 3.**
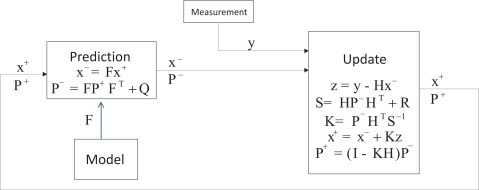
Block diagram of the Kalman filter.

The application of a standard linear Kalman filter requires that the dynamics of the target is represented as a state space model [44]. A simple kinematics approach based on the assumption of the constant velocity process is suggested by some authors, and is shown to track voluntary movements correctly. Figure 3 illustrates the block diagram of the Kalman filter.

The main difference with respect to g–h filters is that a Kalman filter uses covariance noise models for states and observations. A time-dependent estimate of state covariance is updated automatically, and from this the Kalman gain matrix terms are calculated.

### 4.3. Robust Kalman Filter (RKF)

The Kalman filter is commonly used for real-time tracking, but it is not robust to outliers. The sub-movements around the target region caused by motor disorders can be considered as outliers. In our application, it is difficult to define a complete model of the pathological patterns because they are not repetitive or stereotyped. We propose establishing a model of voluntary control and considering the observations that lie outside the pattern of normal distribution as outliers.

**Figure 4.**
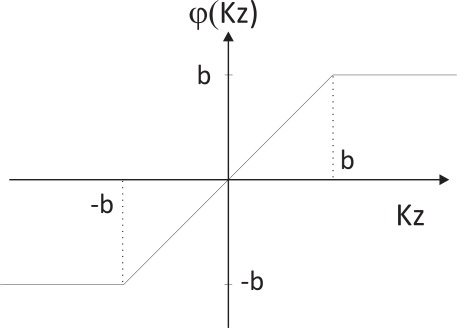
Huber’s function.

The robustification is based on the methodology of the M-estimators following Huber’s function [45]. The difference between the measurement and the estimation is weighted according to Huber’s function. For scalar observations, Huber’s function ψ*_H_* is [46]:

φ(Kz)=Kz⋅min(1,b/|Kz|)

where |*Kz*| is the norm. Figure 4 illustrates Huber’s function.

**Figure 5.**
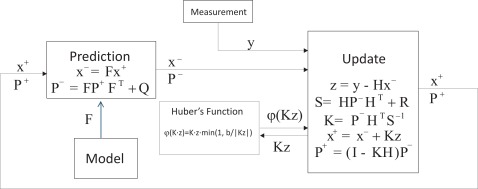
Block diagram of the robust Kalman filter.

Figure 5 depicts the block diagram of the robust Kalman filter. The result is a very easy implementation and simple derivation of the classic Kalman algorithm that includes the detection and elimination of undesirable data by an iterative downweighting of the outlying observations within the least squares method. The selection of the threshold *b* is a trade-off between outlier rejection and control delay. Using this trade-off, *b* was empirically selected (*b* = 5).

### 4.4. Evaluation of the Filtering Techniques

**Figure 6.**
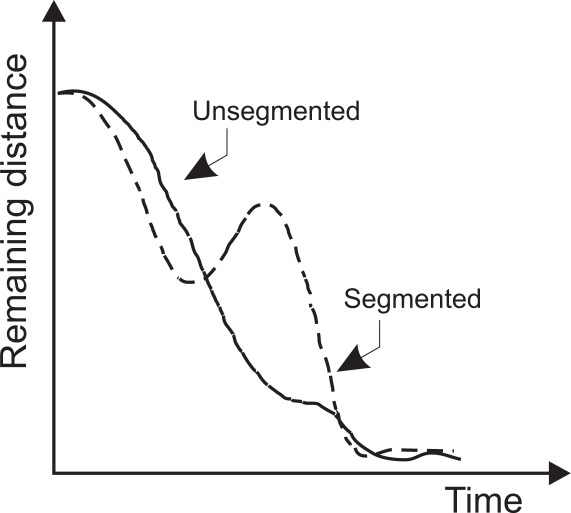
Kinematic descriptor of the improvement introduced by the adaptive filter (movement segmentation).

The filtering algorithms BBF, KF and RKF were applied offline to the previously captured data (Section 3). The performance was compared using a metric called segmentation [47]. Segmentation measures the decomposition of a complex motion into a sequence of simpler movements that are called sub-movements. It is based on extracting maximum distance points during the reaching task. By means of segmentation, overshoots and undershoots can be detected. This metric offers information on the motor performance of both healthy subjects and persons with CP. Figure 6 illustrates an example of segmentation.

The indicators of movement segmentation can be partially interpreted as artifacts that are considered as outliers. Segmentation is estimated calculating the number of local maxima separated by 200 ms from the function “Remaining distance *versus* time”. The mean of sub-movements was M = 1.41 ± 0.18 for the healthy subjects. As expected, the results showed a higher number of sub-movements for the CP subjects. The mean was about 8 sub-movements for CP1, CP2 and CP3 and slightly higher for CP4. Table 5 summarizes the number of sub-movements without and with filters. All filters considerably reduce the number of sub-movements. The RKF reduces movement segmentation up to 65%.

**Figure 7.**
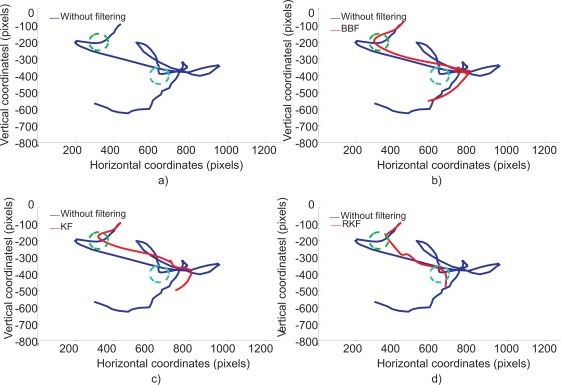
Pointer path performed by CP1 to move the cursor from a target (green circle) to the next target (blue circle). (**a**) without filtering (**b**) with BBF (**c**) with KF (**d**) with RKF.

**Figure 8.**
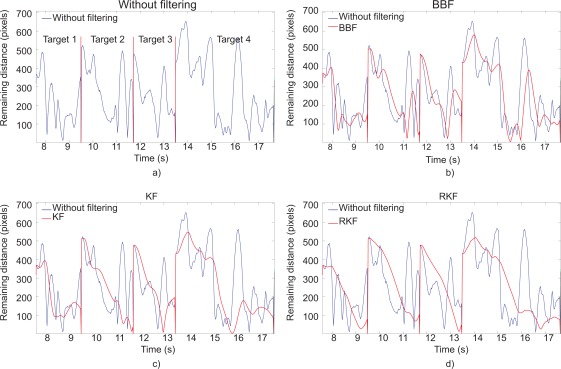
Remaining distance *versus* time for 4 consecutive reaching tasks performed by CP1, (**a**) without filtering, (**b**) with BBF, (**c**) with KF, (**d**) with RKF.

The effect of the filtering techniques can be shown graphically. Figure 7 depicts the target-reaching trajectory without the adaptive filter and with BBF, KF and RKF. Figure 8 shows the remaining distance *versus* time without and with adaptive filters for four consecutive targets. Table 5 shows that the RKF had the best performance followed by the KF and BBF. Although the BBF is able to filter high-frequency movements, it had lower performance than RKF. The reason is that BBF responds faster to changes in movement (involuntary movements) that is undesired. The detection and elimination of outliers (sub-movements following the initial movement), included by the RKF, are more adequate for this application. The gain filter is modulated in real-time and is lower during straight paths in which the prediction error is smaller. By means of outliers suppression and the dynamic gain filter, the initial movement that rapidly covers distance is smoothly filtered, whereas the movements around the target are filtered more strongly. As a consequence, the filtering technique facilitates fine control.

## 5. Evaluation of the Inertial Interface and RKF Algorithm with People with CP

Once the filtering algorithm has been designed, the ENLAZA interface is evaluated as a computer pointing device.

### 5.1. Participants and Methods

The users CP1, CP3 and CP4 participated in this experiment. CP2 was unable to participate because he had left the Cantabria ASPACE center. CP1 usually controls an eye tracking system to use the computer. CP3 and CP4 cannot use any interface (e.g., mouse or keyboard) or even advanced interfaces such as the aforementioned eye tracking system. The users CP3 and CP4 used a pointing magnifier [48]. The experiments and protocols were approved by Cantabria ASPACE expert committee.

The task consisted of reaching the target by clicking on it. The click was performed when the cursor was placed in a region of 60 pixels for 3.5 s. The metric used was the target-reaching time, as a measurement of the acquisition speed. The following three methods were compared:

- Target-reaching task without filter
- Target-reaching task with robust Kalman filter
- Target-reaching task using incremental method

**Figure 9.**
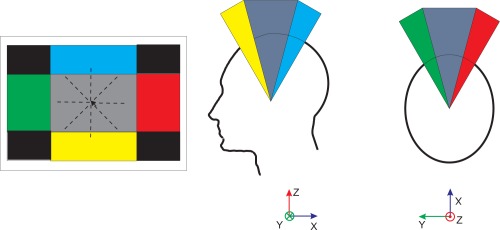
Control space of incremental method. If the user looks at the grey area, the pointer stops. If the user looks at the red region, the pointer moves towards the right.

The incremental method represents a simple way of filtering involuntary movements. In this mode, the pointer increases its position gradually according to the head pose. Figure 9 illustrates this control mode. The experiments were randomized in order to reduce the learning effect (Table 6).

### 5.2. Results

Table 7 summarizes the reaching time for each user and method. The largest difference was observed in CP1. The adaptive RKF reduces the reaching time by a factor of 10. As described in Section 3 the fundamental frequency of CP1 is higher than voluntary movement (Table 3). The filter reduces the effect of high-frequency movements, thus fine control is improved. The reaching time was reduced by a factor of 2 for CP3 and CP4 (hypotonic cases). According to the results in Section 3, correlation with the voluntary motor control was higher for the hypotonic cases. The incremental method also facilitates this control. The RKF presented a better trade-off between reaching and selection than incremental method.

In conclusion, the inertial interface ENLAZA is an effective HCI for people with motor disorders. People who were unable to control conventional interfaces were able to control the computer with an average reaching time between 8 and 18 s. The robust Kalman filter facilitates target acquisition reducing the effect of the involuntary movements on the control.

## 6. Conclusions and Future Work

This work provides the following contributions for the inertial interface. The state-of-the art showed that although there are effective solutions, there is a lack of usability for users with severe motor limitations. The design of the ENLAZA interface demonstrated that inertial technology makes the extraction of pathological patterns possible. These patterns were used to define a user’s needs in terms of motor control, frequency and range of motion. This work provides the following contributions for filtering techniques. A review of the state-of-the-art facilitation techniques for human-computer interaction was presented. The performance of different algorithms to reduce the effects of involuntary movements was studied. As a result, filtering techniques were selected according to the characterization of involuntary movement and posture of people with CP. Finally, a new filtering technique (RKF) based on accurately detecting and reducing deviations in the cursor trajectory was proposed and evaluated. The proposed technique improved fine motor control. Functional evaluation of the ENLAZA interface as a computer pointing device was carried out. Those subjects who were unable to control conventional interfaces were able to control the computer with the ENLAZA interface. Using the RKF algorithm, the reaching time was reduced by a factor of ten for CP1 and two for CP3 and CP4. The results illustrated that the average reaching time ranged between 8 and 18 s. The results and problems that this work faced suggest a field of work that must be addressed in the future. Long-term experiments will be interesting to analyze how physical and cognitive learning affects the device control. The filtering strategy was developed independently of the target location on the screen. As a complement to facilitate the interaction, we will study the application of the filtering strategy with other techniques based on the adaptation of the environment (*i.e.*, click crossing). According to the Cantabria ASPACE team, some users are unable to control eye tracking HCI because of their involuntary movements. Therefore, the RKF will be applied to these alternative interfaces in order to improve the accessibility of alternative HCI. The criterion for the inclusion of participants was the existence of a motor disability that limits possible interaction with assistive products. It will be interesting to extend both the number of users with CP and other groups with similar disabilities (e.g., spinal cord injury or stroke) who often have limited access to the computer. Inertial technology provides a new opportunity for analysis and extraction of kinematic patterns of voluntary and pathological movement. The development of a motion tracking system for full-body analysis is envisaged. The impact of therapies will be evaluated with objective parameters as a complement to the functional and subjective evaluation of the therapists. Motion capture and virtual representation via biofeedback methods motivate users during exercise therapy.

## Figures and Tables

**Table 1.** Usability of advanced interfaces in the literature according to the metric *Throughput*.

**Device**	**Throughput (bits/s)**
Mouse	3.7–4.5
ViewPoint (eyetracker) [31]	2.3–3.7
Touchpad [32]	2.9
GyroPoint (device based on gyroscope) [33]	2.8
**ENLAZA interface**	2
Joystick [32]	1.8
RemotePoint (isometric joystick) [33]	1.4
HeadJoystick [34]	0.92–1.93

**Table 2.** Characteristics of subjects with CP.

**User**	**Motor disorder**		
	**Cervical tone**	**General tone**	**Associated movements**
CP1	Extensor hypertonia	Extensor hypertonia	Athetosis
CP2	Dystonia	Dystonia	Ballistics
CP3	Hypotonia	Hypotonia	No
CP4	Hypotonia	Dystonia	Dystonia

**Table 3.** Maximum frequency (*f_max_*) y 75% (*f*_75_) spectral density (Hz) mean (std).

**User**	***f_max_* (Hz)**			***f*_75%_ (Hz)**		
	**Frontal**	**Sagittal**	**Transverse**	**Frontal**	**Sagittal**	**Transverse**
CP1	1.28 (0.20)	1.59 (0.50)	0.95 (0.30)	3.32 (0.45)	3.70 (0.20)	3.24 (0.26)
CP2	0.39 (0.15)	0.39 (0.18)	0.65 (0.37)	3.31 (0.48)	4.16 (0.32)	3.37 (0.24)
CP3	0.17 (0.16)	0.26 (0.06)	0.39 (0.16)	3.16 (0.38)	1.92 (0.23)	2.39 (0.15)
CP4	0.50 (0.04)	0.325 (0.18)	0.57 (0.11)	3.12 (0.34)	2.43 (0.03)	2.29 (0.17)
HS1	0.35 (0.10)	0.38 (0.04)	0.33 (0.01)	3.52 (0.56)	1.49 (0.15)	1.74 (0.21)
HS2	0.37 (0.09)	0.35 (0.09)	0.33 (0.03)	2.16 (0.24)	1.74 (0.24)	1.72 (0.15)
HS3	0.36 (0.05)	0.39 (0.07)	0.34 (0.06)	2.65 (0.47)	2.68 (0.61)	1.95 (0.17)

**Table 4.** Design principles of the filtering technique based on characterization.

**Features**	**Design principles**
(1) **Heterogeneity**	**Adaptive interface**
	Recognition of the particular user’s needs
(2) **Time domain**	**Enhancement of the fine motor control**
	Reduction of the sub-movements around the target
(3) **Frequency domain**	**Definition of a voluntary control model**
	Filters based on separating frequency bands are not adequate
(4) **Spatial domain**	**Bidimensional filter**
	Independent vertical and horizontal effects

**Table 5.** Mean number of sub-movements per filtering algorithm (std).

**User**	**Filtering algorithms (number of sub-movements (Mean(std)))**			
	**Without filter**	**BBF**	**KF**	**RKF**
CP1	7.92 (1.26)	4.83 (0.96)	3.93 (0.70)	3.5 (0.77)
CP2	8.06 (3.38)	3.97 (1.71)	3.10 (0.98)	2.83 (0.85)
CP3	7.82 (1.54)	4.08 (1.22)	3.04 (0.77)	2.77 (0.76)
CP4	14.35 (8.07)	7.02 (4.09)	4.73 (2.42)	4.64 (2.39)

**Table 6.** Randomized design of the experiments. (**A**) Without filter; (**B**) With RKF; (**C**) With incremental method.

**User**	**Day**				
	**1**	**2**	**3**	**4**	**5**
CP1	CBA	BAC	ABC	ACB	BCA
CP3	BCA	BAC	BCA	BAC	ABC
CP4	BCA	BAC	CBA	ABC	CBA

**Table 7.** Reaching time (seconds) for each user and method.

**User**	**Target reaching time (s). Mean(std)**		
	**Without filter**	**Incremental method**	**RKF**
CP1	109 (10.98)	15.67 (11.70)	8.67 (4.78)
CP3	44.16 (34.77)	19.23 (6.74)	18.08 (14.82)
CP4	43.26 (37.30)	39.97 (21.26)	17.43 (12.20)
